# Inhibitory Effect of *N*,*N*-Didesmethylgrossularine-1 on Inflammatory Cytokine Production in Lipopolysaccharide-Stimulated RAW 264.7 Cells

**DOI:** 10.3390/md7040589

**Published:** 2009-11-17

**Authors:** Taiko Oda, Jong-Soo Lee, Yuta Sato, Yasuaki Kabe, Satoshi Sakamoto, Hiroshi Handa, Remy E. P. Mangindaan, Michio Namikoshi

**Affiliations:** 1 Faculty of Pharmaceutical Sciences, Keio University/Minato-ku, Tokyo 105-8512, Japan; E-Mail: yutasato1125@gmail.com (Y.S.); 2 Department of Natural Products Chemistry, Tohoku Pharmaceutical University/Aoba-ku, Sendai 981-8558, Japan; E-Mails: leejs@gaechuk.gsnu.ac.kr (J.-S.L.); mnami@tohoku-pharm.ac.jp (M.N.); 3 Integrated Research Institute, Graduate School of Bioscience and Biotechnology, Tokyo Institute of Technology/4259 Nagatsuda-cho, Midori-ku, Yokohama 226-8501, Japan; E-Mails: ykabe@bio.titech.ac.jp (Y.K.); ssakamoto@bio.titech.ac.jp (S.S.); handa.h.aa@m.titech.ac.jp (H.H.); 4 Faculty of Fisheries and Marine Science, Sam Ratulangi University/Kampus Bahu, Manado 95115, Indonesia; E-Mail: remysang@yahoo.com (R.E.P.M.)

**Keywords:** LPS, RAW 264.7 cells, NF-κB, TNF-α, *N,N*-didesmethylgrossularine-1, natural product

## Abstract

*N*,*N*-Didesmethylgrossularine-1 (DDMG-1), a compound with a rare α-carboline structure, was isolated from an Indonesian ascidian *Polycarpa aurata* as responsible for the observed inhibitory activity against TNF-α production in lipopolysaccharide-stimulated murine macrophage-like RAW264.7 cells. DDMG-1 inhibited the mRNA level of mTNF-α, IκB-α degradation, and binding of NF-κB to the target DNA site in LPS-stimulated RAW 264.7 cells. Moreover, DDMG-1 had an inhibitory effect on the production of IL-8, which is produced in CD14^+^-THP-1 cells stimulated by LPS. DDMG-1 is thus a promising drug candidate lead compound for the treatment of chronic inflammatory diseases, such as rheumatoid arthritis.

## 1. Introduction

**Figure 1.**
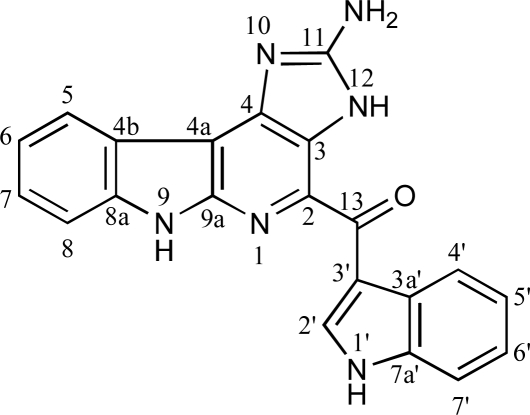
Structure of *N*,*N*-didesmethylgrossularine-1 (DDMG-1).

Macrophages play an important role in immune reactions, allergy and inflammation [1]. By releasing many different kinds of cytokines, these cells induce inflammatory reactions, as well as initiate and maintain specific immune responses. Lipopolysaccharide (LPS), a component of the gram-negative bacterial cell wall, is frequently used as an inflammatory model due to its ability to activate macrophages [2]. In response to microbes and their products such as LPS, macrophages secrete various inflammatory cytokines including interleukin-1 (IL-1), IL-6, chemokines including IL-8, and tumor necrosis factor-α (TNF-α) through the activation of NF-κB [3]. In the case of cancer cachexia, inflammatory cytokines such as IL-6 and TNF-α are considered to be involved, and these cytokines are most likely released from macrophages. In rheumatoid arthritis, a variety of cell populations including macrophages infiltrate the tissue in large numbers, and a range of proinflammatory cytokines are produced abundantly by them. Actually, the generation of CD14-positive macrophage precursors is increased in rheumatoid arthritis [4]. Therefore, the modulation of macrophage-mediated inflammatory responses is important in order to formulate a new therapeutic approach against these inflammatory diseases. In the search for new anti-TNF-α substances, we found that the EtOH extract of an Indonesian ascidian, *Polycarpa aurata*, reduced the production of TNF-α from LPS-stimulated murine macrophage-like RAW264.7 cells. Bioassay-guided isolation yielded *N*,*N*-didesmethylgrossularine-1 (DDMG-1, Figure 1) as an active compound. In the present study, we describe the isolation and biological activity of DDMG-1.

## 2. Results and Discussion

### 2.1. Confirmation of DDMG-1

DDMG-1 was obtained as a yellow pigment from *P. aurata* collected from a coral reef in the Lembeh Strait, North Sulawesi, Indonesia. The EI mass spectrum of DDMG-1 showed a molecular ion at *m*/*z* 366, and the molecular formula C_21_H_14_N_6_O was deduced from EIMS and NMR spectra. The ^1^H-NMR spectrum of DDMG-1 revealed the presence of nine aromatic protons, and a 3-substituted indole moiety that was assigned based on the characteristic signals at δ 9.34 (H-2′), 8.47 (H-4′), 7.48 (H-7′), and 7.23 (H-5′ and 6′). The remaining signals were ascribed to four hydrogen atoms on another indole (α-carboline) moiety.

^1^H- and ^13^C-NMR data suggested that this compound is an α-carboline alkaloid possessing an indole ring as a side chain. A literature search yielded the structure of DDMG-1 as a candidate, and this structure was confirmed by comparing the ^13^C-NMR data with the reported values [5] and ^1^H- and ^13^C-NMR spectra with the Supplemental Information provided in a report on the synthesis of DDMG-1 and grossularine-1 [6]. Since DDMG-1 has a unique α-carboline structure and considering the fact that this was the first study to identify the anti-TNF-α activity of α-carboline alkaloids, the biological activity of DDMG-1 in the production of murine and human inflammatory cytokines was further studied.

### 2.2. DDMG-1 inhibited LPS-induced mTNF-α and mIL-6 production in RAW 264.7 cells

**Figure 2.**
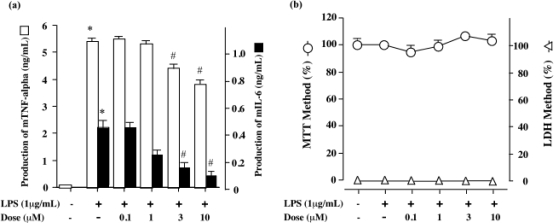
Effects of DDMG-1 on mTNF-α and mIL-6 production and cell proliferation induced by LPS-stimulated RAW 264.7 cells. (a) Effects of DDMG-1 on mTNF-α and mIL-6 production by LPS-stimulated or non-stimulated RAW264.7 cells. RAW 264.7 cells (1 × 10^6^ cells/mL) were untreated or treated with LPS (1 μ g/mL) with the indicated concentrations of DDMG-1 for 24 h, and mTNF-α and mIL-6 levels in the culture supernatant were measured by ELISA, as described in Materials and Methods. This experiment was repeated three times on different days. Figures were created based on the results. *p < 0.05 *vs*. Control; ^#^p < 0.05 *vs*. LPS. (b) Effects of DDMG-1 on cell proliferation. The MTT assay and LDH data were obtained as described in Materials and Methods. Data are the means relative (%) to the control value.

mTNF-α and mIL-6 concentrations in the culture supernatants under each experimental condition in LPS-stimulated RAW 264.7 cells were determined by ELISA. RAW 264.7 cells in an unstimulated state released 0.4 ng/mL TNF-α into the culture media during incubation for 24 h, whereas the cells showed marked TNF-α production on exposure to LPS alone (Figure 2a). DDMG-1 inhibited LPS-induced TNF-α production in a dose-dependent manner (Figure 2a). RAW 264.7 cells in a normal state released 90 pg/mL mIL-6 into the culture medium during incubation for 24 h, whereas the cells showed a marked increase in mIL-6 production up to 410 pg/mL upon exposure to LPS alone (Figure 2a). DDMG-1 inhibited LPS-induced mIL-6 production in a dose-dependent manner (Figure 2a). In order to elucidate whether DDMG-1 at effective concentrations showed toxicity against RAW 264.7 cells, we employed the MTT and LDH methods. The decreased MTT value reflects cytotoxicity and the inhibitory effect on cell proliferation. However, these cannot be distinguished. We detected cytotoxicity using LDH assays. Data on LDH assay were showed to Figure 2b. The results indicated that the inhibiton of TNF-α production by DDMG-1 was not attributable to its nonspecific cell toxicity (Figure 2b). Because the production of TNF-α was greater than that of mIL-6 under LPS-stimulated conditions, we subsequently planned to use a TNF-α production system.

### 2.3. DDMG-1 down-regulated TNF-a mRNA expression at the transcriptional level

**Figure 3.**
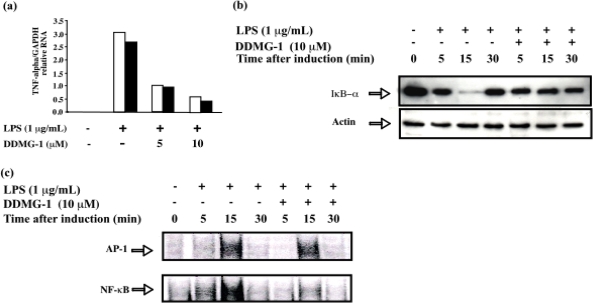
Effects of DDMG-1 on intracellular signaling molecules in LPS-stimulated RAW 264.7 cells. (a) Effect of DDMG-1 on the LPS-induced synthesis of TNF-α transcripts. RAW 264.7 cells were pretreated with DDMG-1 for 1 h and stimulated with LPS for 4 hr. Total cellular RNA was subjected to real-time PCR. The relative ratio as a percentage is also shown, whereby the TNF-α signal was normalized to the GAPDH signal. We conducted two independent experiments and graphically presented the results separately (□, ▪). (b) Effects of DDMG-1 on NF-κB transcription by IκB-α̣ degradation. Western blotting of RAW 264.7 cells treated with LPS and DDMG-1, as indicated. Total proteins were extracted at the indicated times, resolved by 10% SDS-PAGE, blotted, and examined by Western blotting using polyclonal anti-serum against IκB-α. (c) Effects of DDMG-1 on the DNA-binding activities of NF-κB and AP-1 transcription factors. RAW 264.7 cells were treated with LPS and DDMG-1 as indicated, followed by nuclear extraction. EMSA was performed as described in Materials and Methods using ^32^P-labeled probes that possessed NF-κB- and AP-1-binding sites. The NF-κB/probe and AP-1/probe complex are indicated.

To clarify whether DDMG-1 affects TNF-α expression, semiquantitative RT-PCR was carried out using the total RNA of LPS-stimulated RAW 264.7 cells. TNF-α mRNA expression markedly increased when cells were simulated with LPS alone. Within 24 h after LPS stimulation, the highest expression was observed at 4 h. Thus, the effect of DDMG-1 on TNF-α expression was examined at 4 h. The results are shown in Figure 3a. Since two independent experiments showed the almost same results and the significant difference was observed between control and test experiments, third experiment was not performed and the data of two experiments are shown in Figure 3a. DDMG-1 attenuated the LPS-induced synthesis of TNF-α mRNA expression in a dose-dependent manner; however, levels of housekeeping GAPDH transcripts remained unchanged on the treatment of RAW 264.7 cells with LPS and/or DDMG-1. The above results indicate that DDMG-1 inhibited TNF-α production at the mRNA level in LPS-stimulated RAW 264.7 cells.

### 2.4. DDMG-1 inhibited IκB-α degradation in LPS-stimulated RAW 264.7 cells

To investigate whether the inhibitory action of DDMG-1 is attributable to its sequential effect on IκBα degradation, Western blot analysis was carried out with cytoplasmic extracts of LPS-stimulated RAW 264.7 cells. Upon exposure to LPS alone, cellular IκBα was markedly degraded at 15 min (Figure 3b). DDMG-1 showed a significant inhibitory effect on LPS-induced IκB-α degradation in the time-course study (Figure 3b). Moreover, we examined the effects of DDMG-1 on MAPK activation in LPS-stimulated RAW 264.7 cells, but DDMG-1 did not affect MAPK activation (data not shown).

### 2.5. DDMG-1 inhibited LPS-stimulated binding of NF-κB to DNA

DDMG-1 showed a significant inhibitory effect on LPS-induced IκBα degradation in the time-course study; therefore, it is expected that DDMG-1 inhibits the LPS-stimulated binding of NF-κB to target DNA. EMSA was performed using the nuclear extract, and inhibition was observed as expected (Figure 3c). The results indicated that DDMG-1 specifically inhibited the binding of NF-κB to the target DNA site.

### 2.6. DDMG-1 inhibited LPS-induced hTNF-α hIL-1b, hIL-6, and IL-8 production in CD14^+^-THP-1 cells

**Figure 4.**
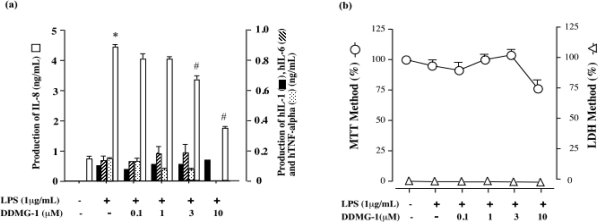
Effects of DDMG-1 on hTNF-α, hIL-8, hIL-6, and hIL-1β production and cell proliferation induced by LPS-stimulated CD14^+^-THP-1 cells. (a) Effects of DDMG-1 on hTNF-α, hIL-8, hIL-6, and hIL-1β production in LPS-stimulated or non-stimulated CD14^+^-THP-1 cells. CD14^+^-THP-1 cells (1 × 10^6^ cells/mL) were untreated or treated with LPS (1 μg/mL) at the indicated concentrations of compounds for 24 h. hTNF-α, hIL-8, hIL-6, and hIL-1β levels in the culture supernatant were measured by ELISA, as described in Materials and Methods. This experiment was repeated three times on different days. Figures were created based on the results. *p < 0.05 *vs*. Control; ^#^p < 0.05 *vs*. LPS. (b) Effects of DDMG-1 on cell proliferation. MTT and LDH assays were conducted as described in Materials and Methods. Data are the mean values relative (%) to the control.

As DDMG-1 inhibited mTNF-α and mIL-6 production in LPS-stimulated RAW 264.7 cells, we examined whether DDMG-1 inhibited cytokine production in LPS-stimulated CD14^+^-THP-1 cells. Recently, it was clarified that THP-1 cells do not produce IL-8 under LPS- and PMA-stimulated conditions. On the other hand, CD14^+^-THP-1 cells produced a high concentration of IL-8 under LPS-stimulated conditions. Therefore, we tested whether DDMG-1 dose-dependently inhibited IL-8 production in LPS-stimulated CD14^+^-THP-1 cells. The results indicated that DDMG-1 inhibited IL-8 production by LPS-stimulated CD14^+^-THP-1 cells (Figure 4a). Moreover, we examined whether DDMG-1 inhibits hTNF-α, hIL-1β, and hIL-6 production under the same experimental conditions as for IL-8 production. The results shown in Figure 4a revealed that hTNF-α, hIL-1β, and hIL-6 production was lower than that of IL-8, and DDMG-1 (10 μM) inhibited hTNF-α and hIL-6 production, but the effect of DDMG-1 on IL-1β production was not determined. Evaluation of the influences of MTT solution on the cell survival rate revealed a decrease at a concentration of 10 μM, but we did not observe cell death using the LDH method (Figure 4b). Specifically, an inhibitory effect on cell proliferation was observed at a high concentration of DDMG-1. The result was judged not as cytotoxicity but as an inhibitory effect on cell proliferation. Only small amounts of hTNF-α, hIL-6, and hIL-1β were produced in this experimental system. The experimental data to examine the effect of DDMG-1 lack accuracy. The inhibitory effect of DDMG-1 seems to be weak.

Three α-carboline alkaloids, grossularines-1 and -2 [7] and DDMG-1 [5], have thus far been isolated from ascidians. Grossularines-1 and -2 showed weak cytotoxicity against murine leukemia L1210 cells and were more cytotoxic to human colon (WiDr) and breast (MCF7) tumor cell lines [7,8]. The cytotoxic mechanisms of the two compounds were suggested to be different [8]. On the other hand, the biological activity of DDMG-1 has not been reported. DDMG-1 exhibited modest inhibitory activity against the colony formation of Chinese hamster V79 cells (EC_50_ = 10 μM) [9], and, in this study, we identified a new bioactivity in which DDMG-1 inhibited the production of mTNF-α from LPS-stimulated RAW264.7 cells by inhibiting IκB-α degradation and NF-κB binding to the target DNA Moreover, we demonstrated that IL-8 in the culture supernatant of LPS-stimulated CD14^+^-THP-1 cells was also decreased by DDMG-1 treatment.

The overexpression of TNF-α has been detected in chronic inflammatory diseases and, therefore, compounds that control the production of TNF-α will become drug candidates for the treatment of inflammatory diseases [10,11]. In rheumatoid arthritis, a variety of cell populations including macrophages infiltrate the tissue in large numbers, and a range of proinflammatory cytokines [IL-1, IL-6, and IL-8] are abundantly produced by them [12]. Moreover, it has been said that even tumorigenesis is caused by macrophage activation. Apart from these types of inflammation, macrophages are often observed in solid tumors, where they are called tumor-associated macrophages [13]. NF-κB is a transcription factor that promotes the transcription of genes of many proinflammatory cytokines. In macrophages, it is considered to regulate the production of IL-1, IL-6, IL-8, and TNF-α. NF-κB is activated by extracellular signals such as LPS, IL-1, and TNF-α. Besides the existing activation route for NF-κB, a nonclassical pathway has recently been identified [14]. Our experiment using RAW264.7 cells demonstrated that, in TNF-α induction accompanied by activated NF-κB under LPS stimulation, reduced TNF-α in the culture supernatant due to DDMG-1 suppressed IκBα degradation, with resulting decreases in both the binding capacity of NF-κB to the target DNA and mRNA expression. Thus, DDMG-1 was demonstrated to reduce the inducibility. However, this study does not reveal whether the specific effect of DDMG-1 on NF-κB signaling is exerted directly or indirectly. In this regard, data from other experimental systems, such as a study on the effects of different stimuli using the same cell, are needed. Although the inhibitory activity of DDMG-1 against TNF-α production is not significant, DDMG-1, with its rare α-carboline structure, is a promising lead compound for the development of anti-TNF-α drugs.

## 3. Experimental Section

### 3.1. General

^1^H- and ^13^C-NMR spectra were recorded on a JEOL JNM-AL-400 NMR spectrometer at 400 MHz for ^1^H and 100 MHz for ^13^C in CD_3_OD (δ_H_ 3.30, δ_C_ 49.0). Mass spectra were obtained by a JEOL JMS-MS 700 mass spectrometer (EI mode).

### 3.2. Isolation of DDMG-1

The solitary ascidian *P. aurata* (2.2 kg, wet weight) was collected by scuba diving (−3~7 m) in the Lembeh Strait, Indonesia, in 2008, cut into small pieces, and soaked in ethanol. The ethanol extract was evaporated and the residue was dissolved in MeOH-H_2_O (4:1) and washed with *n*-hexane. The methanolic solution was concentrated and diluted with water to a MeOH content of 40%, and the solution was extracted with CHCl_3_. The CHCl_3_ extract (12.3 g) was subjected to chromatography on SiO_2_ with CHCl_3_-MeOH (gradient elution) to give six fractions. The fourth fraction (7:3 elution, 8.0 g) was separated by an ODS column (50, 70, 85, and 100% MeOH) into four fractions. The second fraction (70% MeOH elution, 0.6 g) was purified by HPLC (ODS, 70% MeOH) to yield 34.4 mg of DDMG-1. The structure of DDMG-1 is shown in Figure 1.

*DDMG-1*: yellow pigment; ^1^H-NMR (CD_3_OD) δ 9.34 (1H, s, H-2′), 8.47 (1H, m, H-4′), 8.23 (1H, d, *J* = 8.0 Hz, H-5), 7.48 (2H, m, H-8 and H-7′), 7.41 (1H, dd, *J* = 8.0, 8.0 Hz, H-7), 7.23 (3H, m, H-6, H-5′, and H-6′); ^13^C-NMR (CD_3_OD) δ 187.3 (C-13), 153.4 (C-11), 148.8 (C-2), 141.8 (C-9), 138.9 (C-2′), 137.5 (C-8a), 135.9 (C-7a′), 131.8 (C-3), 128.9 (C-7), 128.7 (C-4), 124.3 (C-3a′), 123.7 (C-5), 123.3 (C-4′), 123.2 (C-6′), 122.6 (C-5′), 121.3 (C-4b), 118.8 (C-6), 115.8 (C-3′), 112.8 (C-7′), 112.4 (C-8), 103.5 (C-4a); EIMS *m*/*z* 366 (M^+^, 100%), 249 [(M – indole)^+^], 221 [(M – indole-CO)^+^].

### 3.3. Materials

Fetal bovine serum (FBS) and other culture materials were purchased from Invitrogen (Carlsbad, CA, USA). Anti-actin was purchased from Santa Cruz Biotechnology (Santa Cruz, CA, USA). All other chemicals, including endotoxin LPS (*Escherichia coli 055-85*) and 3-(4,5-dimethylthiazol-2-yl)-2,5-diphenyltetrazolium bromide (MTT), were purchased from Sigma-Aldrich (St. Louis, MO, USA).

### 3.4. Cell culture

RAW 264.7 cells were grown in culture medium (RPMI medium, containing 10% FBS, 2 mM glutamine, 100 U/mL of penicillin G and 100 μg/mL of streptomycin). THP1-Blue™-CD14 (CD14^+^-THP-1) cells were purchased from InvivoGen. The cells were maintained and subcultured in growth medium (RPMI medium) supplemented with 200 μg/mL Zeocin™ and 10 μg/mL Blasticidin. These cells were cultured in humidified (5% CO_2_) incubator at 37 °C.

### 3.5. Murine TNF-α (mTNF-α), murine IL-6 (mIL-6), human TNF-α (hTNF-α), human IL-β (hIL-β), human IL-6 (hIL-6), and human IL-8 (hIL-8) quantification

RAW 264.7 cells or CD14^+^-THP-1 cells were pretreated with DDMG-1 for 5 min. After LPS (final concentration: 1 μg/mL) was added, except to the control, experimental conditional cells were cultured for 24 h at 37 °C. The inflammatory cytokine concentration of each culture supernatant was measured using an ELISA kit following the manufacturer’s instructions (R&D Systems, Inc., Minneapolis, MN, USA).

### 3.6. Measurement of TNF-α mRNA expression

RAW264.7 cells (3 × 10^5^ cells/35 mm dishes) were incubated with 5 or 10 μM of DDMG-1 for 1 h, and then LPS(1 μg/mL) was added. After incubation for 4 hr, the cells were recovered and washed with PBS(-) twice. This is because the mRNA expression level was the highest at 4 h after stimulation.

Total RNA was extracted with Sepasol-RNA I (Nacalai Tesque Co., Kyoto, Japan). SuperScript III (Invitrogen) was used to synthesize first-strand cDNAs from 1 μg total RNA using oligo-dT_15_ primers. PCR was performed using the QuantiTect SYBER Green PCR Master Mix (TOYOBO Co.) with an ABI 7300 real-time PCR system (Applied Biosystems). Primers for quantitative PCR (Q-PCR) were as follows: TNF-α sense primer: 5′-CCAAGGACACCCCTGAGGGGGC-3′, TNF-α antisense primer: 3′-TCACAGAGCAATGACTCTTGCCCG-5′, GAPDH sense primer; 5′-TTACTCCTTGGAGGCC-ATGTAG-3′, GAPDH antisense primer: 3′-AATGACAACTTTGTCAAGCTCATT-5′. A thermal cycle of 95 °C, 15 s; 60 °C, 30 s; and 72 °C, 30 s was repeated for 40 cycles.

### 3.7. Western blot analysis

RAW 264.7 cells were pretreated with DDMG-1 for 5 min and stimulated with LPS (1 μg/mL) for 5–30 min. Cytoplasmic extracts of cells were subjected to Western blot analysis with anti-IκB antibody. The blots were finally reacted with ECL reagent (Amersham-Pharmacia, San Francisco, USA) and exposed to X-ray film [15].

### 3.8. Cell viability assay and determination of cytotoxicity

Lethal cell injury was assessed by measuring lactate dehydrogenase (LDH) release [16], while proliferation was evaluated by enumerating viable cells using the MTT formazan production method [17]. RAW 264.7 cells were incubated with various concentrations (0.1, 1, 3, and 10 μM) of DDMG-1 for 24 h. The cells were treated with MTT solution and, after incubation for 3h, formazan production was assessed by measuring the OD (570 nm).

### 3.9. Preparation of nuclear extracts and EMSA

Nuclear extracts were prepared from RAW 264.7, cells as described previously [15], and aliquots were frozen at −80 °C. EMSA was conducted on 5% polyacrylamide gels in 1 × Tris-borate/EDTA electrophoresis buffer. mTNF-α/NFκ-B and AP-1 were synthesized by Rikaken (Aichi, Japan). The other experimental conditions were as described previously [15,18].

### 3.10. Statistical analysis

Each experiment was performed at least three times, and representative data are shown. Means were checked for significant differences using Student’s *t*-test, with p-values of <0.05.

## 4. Conclusions

DDMG-1 inhibited the mRNA level of mTNF-α, IκB-α degradation, and binding of NF-κB to the target DNA site in LPS-stimulated RAW 264.7 cells. Moreover, DDMG-1 exhibited an inhibitory effect on IL-8 production, which is produced in CD14^+^-THP-1 cells stimulated with LPS. DDMG-1, which has a rare α-carboline structure, is thus a promising drug candidates lead of for the treatment of chronic inflammatory diseases, such as rheumatoid arthritis.
